# Breakdown of the central synapses in C9orf72-linked ALS/FTD

**DOI:** 10.3389/fnmol.2022.1005112

**Published:** 2022-09-16

**Authors:** Layla T. Ghaffari, Davide Trotti, Aaron R. Haeusler, Brigid K. Jensen

**Affiliations:** Jefferson Weinberg ALS Center, Department of Neuroscience, Vickie and Jack Farber Institute for Neuroscience, Thomas Jefferson University, Philadelphia, PA, United States

**Keywords:** amyotrophic lateral sclerosis, frontotemporal dementia, synaptic dysfunction, neurodegenerative disease, C9orf72

## Abstract

Amyotrophic lateral sclerosis (ALS) is a progressive, fatal neurodegenerative disease that leads to the death of motor and cortical neurons. The clinical manifestations of ALS are heterogenous, and efficacious treatments to significantly slow the progression of the disease are lacking. Cortical hyper-excitability is observed pre-symptomatically across disease-causative genetic variants, as well as in the early stages of sporadic ALS, and typically precedes motor neuron involvement and overt neurodegeneration. The causes of cortical hyper-excitability are not yet fully understood but is mainly agreed to be an early event. The identification of the nucleotide repeat expansion (GGGGCC)_n_ in the *C9ORF72* gene has provided evidence that ALS and another neurodegenerative disease, frontotemporal dementia (FTD), are part of a disease spectrum with common genetic origins. ALS and FTD are diseases in which synaptic dysfunction is reported throughout disease onset and stages of progression. It has become apparent that ALS/FTD-causative genes, such as *C9ORF72*, may have roles in maintaining the normal physiology of the synapse, as mutations in these genes often manifest in synaptic dysfunction. Here we review the dysfunctions of the central nervous system synapses associated with the nucleotide repeat expansion in *C9ORF72* observed in patients, organismal, and cellular models of ALS and FTD.

## Introduction

Amyotrophic lateral sclerosis (ALS) is a progressive, fatal neurodegenerative disease that leads to the death of motor neurons. The clinical manifestations of ALS are heterogenous (Taylor et al., 2016; Abramzon et al., 2020; Bendotti et al., 2020; Masrori and Van Damme, 2020), and efficacious treatments to significantly slow the progression of the disease are lacking. Identification of genetic risk factors has provided evidence that ALS and another neurodegenerative disease, frontotemporal dementia (FTD), are part of a disease spectrum with common genetic origins. One such causative genetic risk factor is the aberrant intronic (GGGGCC)_n_ nucleotide repeat expansion (NRE) in the *C9ORF72* gene (C9). The C9-NRE has been identified as the most common genetic cause of ALS and FTD, and individuals with the C9-NRE can have ALS, FTD, or both (Dejesus-Hernandez et al., 2011; Renton et al., 2011). The NRE is suspected of leading to neurodegeneration in three ways that may not be mutually exclusive. The C9-NRE DNA can be transcribed into repeat-containing RNA, which can form secondary structures such as hair-pins and G-quadruplexes and thus sequester RNA-binding proteins critical for cell survival (Donnelly et al., 2013; Haeusler et al., 2014; Burguete et al., 2015; Conlon et al., 2016). Five distinct dipeptide repeat protein species (DPRs); poly(GA), poly(GR), poly(GP), poly(PA), and poly(PR) are unconventionally produced through repeat-associated non-AUG (RAN) translation of the pathogenic C9-NRE RNA in both sense and antisense directions (Mori et al., 2013; Zu et al., 2013) and confer neurotoxicity (Wen et al., 2014, 2017; Freibaum and Taylor, 2017). Thirdly, there is loss of C9ORF72 RNA and protein levels due to the transcription-repressive effect of the C9-NRE (Dejesus-Hernandez et al., 2011; Donnelly et al., 2013). Other genetic risk factors on the ALS-FTD disease spectrum include mutations in DNA/RNA-binding proteins TAR DNA-binding protein 43 (*TARDBP, TDP-43*) and fused in sarcoma (*FUS*), both of which are predominantly nuclear proteins but have recently been found to have a function at the synapse (Ling, 2018). When mutated, FUS and TDP-43 are frequently found in cytoplasmic inclusions and/or are lost from the nucleus in patient postmortem tissue (Dormann and Haass, 2011). TDP-43 is further implicated in the pathogenesis of ALS and FTD, as loss of nuclear TDP-43 and cytoplasmic aggregates are the most common pathological hallmarks of both sporadic and familial ALS and FTD in postmortem tissues (Suk and Rousseaux, 2020). Genetic variations in the superoxide dismutase 1 gene (*SOD1*), involved in the cellular antioxidant defense and homeostasis of reactive oxygen species (Bowling et al., 1993; Rosen et al., 1993), are other risk factors for ALS. Apart from SOD1 localization in mitochondria, which are found at synapses, SOD1 has not been reported to have a specific function at the synapse. Still, the loss of function and misfolding of the encoded mutant SOD1 protein leads to synaptic dysfunction (Guo et al., 2000; Magrane et al., 2012; Fogarty, 2019).

The synapse is the meeting place of communication between neurons. Synaptic communication breakdown is a shared phenotype of neurological diseases (Sudhof and Malenka, 2008; Lepeta et al., 2016). Neuron-to-neuron synapses are composed of a pre-synaptic and post-synaptic compartment, each with unique proteins and structures to facilitate excitatory and inhibitory neurotransmission. The neuromuscular junction (NMJ) is the synapse between a motor neuron and muscle fibers. It resembles a neuron-neuron synapse, with the pre-synaptic compartment being the motor neuron terminal and the post-synaptic compartment being a muscle cell. The NMJs are ultimately degenerating in all the forms of ALS, including ALS/FTD, however, here we review the dysfunctions of the central nervous system synapses associated with the C9-NRE observed in patients, organismal, and cellular models of ALS and FTD. Furthermore, neuronal activity controls proper neuronal development, synaptic function, and ultimately health and survival of the individuals and organisms (Flavell and Greenberg, 2008; Turrigiano, 2012; Yap and Greenberg, 2018). Activity-dependent pathways are essential for adaptivity to external stimuli, whereas defects in components of these pathways are associated with neurological disorders (Yap and Greenberg, 2018).

ALS and FTD are diseases in which synaptic dysfunction is reported throughout disease onset and stages of progression. Synaptic dysfunction is an umbrella term that encompasses alterations in the morphology of neurons at dendritic spines or dendritic arborization, changes in electrophysiological properties, such as amplitude or frequency of action potential firing, membrane capacitance or resting membrane potential, mishandling of intracellular calcium signaling, and modulation of levels of synaptic proteins. Deficits in synaptic structure, function, and synaptic protein levels are well-documented in neurological diseases (Lepeta et al., 2016; Bae and Kim, 2017). It has become more apparent that ALS/FTD-causative genes, such as *C9ORF72*, may have roles in maintaining the normal physiology of the synapse, as mutations in these genes often manifest in synaptic dysfunction. For example, it is thought that the C9-NRE can alter the neuronal excitability (Sareen et al., 2013; Devlin et al., 2015; Starr and Sattler, 2018) and thus could trigger a feed-forward loop of activity-dependent dysfunctions. While there are few studies related to overt changes in excitability in FTD patients, it has been shown that deficits in functional neuronal networks occur (Lee et al., 2014; Huber et al., 2021). Related to ALS, cortical hyper-excitability is observed pre-symptomatically across disease-causative genetic variants, as well as in the early stages of sporadic ALS, and typically precedes motor neuron involvement and overt neurodegeneration (Vucic et al., 2008; Bae et al., 2013; Geevasinga et al., 2015, 2016; Wainger and Cudkowicz, 2015; Walhout et al., 2015; Schanz et al., 2016; Brunet et al., 2020). The causes of cortical hyper-excitability are not yet fully understood but is mainly agreed to be an early event in disease progression. Still, there is evidence that suggests an excitatory-inhibitory imbalance. Notably, modulation of neuronal activity in ALS is a primary topic of interest, and the first FDA-approved drug for ALS, Riluzole, was shown to dampen neuronal activity (Bellingham, 2011). Moreover, alterations in glutamatergic neurotransmission have been identified as a source of neuronal stress in ALS (Rosenblum and Trotti, 2017; Armada-Moreira et al., 2020). Excess glutamate in the CSF of ALS and FTD patients has been reported (Rothstein et al., 1990; Palese et al., 2020), in addition to changes in glutamate receptor subunits' composition and function, which are described in this review.

## Clinical features of central synaptic dysfunction in patients and patient samples

**Figure 1:**
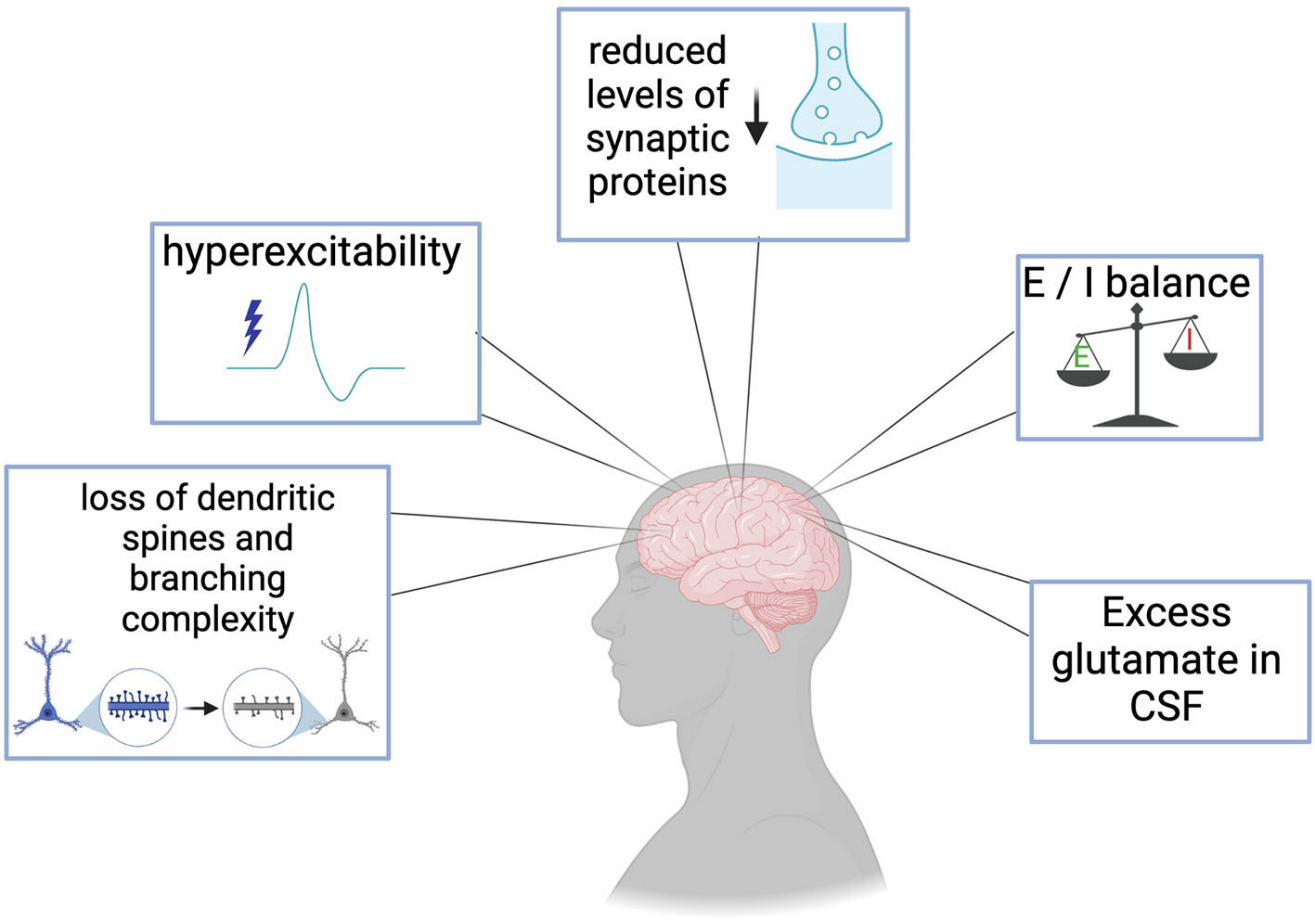
Clinical manifestations and postmortem evidence of synaptic dysfunction in ALS and FTD include loss of dendritic spines and branching complexity in cortical regions, cortical hyperexcitability prior to the loss of motor neurons, reduction in essential synaptic proteins such as SV2a, imbalances in excitatory and inhibitory systems, and excess glutamate in patient cerebrospinal fluid (CSF).

Clinical evaluation of muscle and neuronal excitability levels as well as measurement of neurotransmitter systems in the central nervous system (CNS) of ALS and FTD patients has yielded critical information regarding the timeline of excitability events throughout disease progression and has recently been reviewed elsewhere (Gunes et al., 2020). Changes in excitability have sparked interest in ALS and FTD for several years. Published studies in postmortem tissues have provided valuable clues on the role of neurotransmitter systems, ion channel dysfunctions, synaptic protein alterations, network connectivity, and morphological assessments in the pathogenesis of these diseases. The knowledge has shaped the direction of basic and translational ALS/FTD research. Figure 1 and Table 1 summarizes evidence of the broad central synaptic dysfunction observed in ALS and FTD.

**Table 1:** Evidence of synaptic dysfunctions observed in ALS and FTD patients and postmortem tissue.

	**ALS**	**FTD**
Excitatory system	• Excess glutamate in CSF (Rothstein et al., 1990; Pioro et al., 1999) • Deficient glutamate uptake in the motor cortex, somatosensory cortex, and spinal cord (Rothstein et al., 1992; Bristol and Rothstein, 1996) • Loss of the excitatory amino acid transporter 2 (EAAT2) in postmortem motor cortex (Bristol and Rothstein, 1996) • Downregulation of GluN1, GluN2a, GluN2D, GluA2, and GluA3 transcripts (Aronica et al., 2015) • Upregulation of GluA1, GluN2A, and GluN2B in the frontal cortex, and gene clusters related to neuroinflammation in the spinal cord (Andres-Benito et al., 2017)	• Autoantibodies to GluA3 in the CSF of FTLD-tau patients and reduction of the GluA3 protein in the temporal cortex (Palese et al., 2020) • Increase in GluA2 subunit protein (Palese et al., 2020) • Reduction in intracortical facilitation in patients with GluA3 autoantibodies (Palese et al., 2020) • Increase in serine and glutamate in CSF of FTD-patients (Palese et al., 2020)
Inhibitory system	• Loss of parvalbumin interneurons (Nihei et al., 1993) • Reduction in GABAergic inhibition in the motor cortex (Turner et al., 2005; Foerster et al., 2012) • Reduction of GABAα1 transcripts in postmortem motor, frontal, and temporal cortices (Petri et al., 2006) • Elevated GABAβ1 subunit mRNA in the motor cortex, and mRNA for the enzyme glutamic acid decarboxylase (GAD) is elevated in frontal and temporal cortices (Petri et al., 2003, 2006) • Upregulation of GABBRA1, GABBR2 in frontal cortex (Andres-Benito et al., 2017)	• TMS studies indicate deficits in the inhibitory system in FTD (Benussi et al., 2017)
Synaptic proteins	• Decreased levels of the trans-synaptic organizer neuronal pentraxin receptor (NPTXR) in C9-FTD vs. C9-NRE carriers, which is a trans-synaptic organizer of excitatory and inhibitory synapses (Barschke et al., 2020) • Pre-symptomatic C9-NRE mutation carriers, PET imaging with [^11^C] UCB-J, which binds to the synaptic vesicle protein, SV2A, revealed a synaptic loss in the thalamus before symptom onset (Malpetti et al., 2021)	• Neuronally derived exosomes isolated from FTD patient plasma have reduced levels of synaptotagmin, synaptophysin, and neurogranin, and increase of pre-synaptic synapsin-1 protein (Goetzl et al., 2016)
Morphological defects	• Apical dendrites, in addition to the cell bodies of Betz cells in layer V degenerate in the cortex of ALS patients (Udaka et al., 1986) • Apical dendrite degeneration of Betz cells in fALS, sALS, and FTD-ALS (Genc et al., 2017) • Significant reduction in the number of synapses in motor cortex (Genc et al., 2017) • Loss of synapses in pre-frontal cortex correlating with severity of cognitive impairment (Henstridge et al., 2018) • Selective loss of tripartite synapses in the spinal cord of C9-NRE patients (Broadhead et al., 2022)	• Decreased density of dendritic spines on layer III pyramidal cells in the postmortem cortex (Catala et al., 1988; Ferrer, 1999), • Degeneration of dendrites of the dentate gyrus (Terreros-Roncal et al., 2019) • Severe reduction in dendritic arborization and spine density in upper cortical layers (Ferrer et al., 1991)
Functional studies	• Cortical hyperexcitability is observed in ALS patients (Eisen et al., 1993; Kanai et al., 2006; Vucic and Kiernan, 2006; Vucic et al., 2008; Bae et al., 2013; Geevasinga et al., 2015, 2016; Wainger and Cudkowicz, 2015; Walhout et al., 2015; Schanz et al., 2016; Brunet et al., 2020; Menon et al., 2020).	• Hyperexcitability is yet not observed in the C9-FTD patient populations (Schanz et al., 2016)

These dysfunctions include defects in excitatory and inhibitory neurotransmitter systems, and changes in synaptic protein levels.

## Excitability and synaptic activity

Altered synaptic activity and neuronal network excitability have been reported in ALS/FTD patient-derived neurons and in C9-NRE-expressing *in vivo* models, DPR-expressing models, and C9orf72 ablation models. Reported synaptic dysfunction phenotypes vary and seem to be dependent upon cell type, and model age (*in vitro* or *in vivo*). The advent of induced pluripotent stem cells (iPSC) has allowed the ALS and FTD fields to investigate disease-relevant molecular pathways on the genetic background of patients, including the presence of the NRE. These models have paved the way for mechanistic analyses of temporal synaptic dysfunction *in vitro*, and most reported synaptic dysfunctions are within these models. Moreover, emerging technologies such as multi-electrode array (MEA) have since become the gold standard for studying excitability *in vitro* and have made probing the underpinnings of excitability more accessible. There is variation in the disease phenotypes among the different iPSC models, possibly due to numerous factors that may include: differentiation protocols, duration of culture, reagents, purity of cultures, and origin of iPSC lines. The phenotypic variation observed in iPSC models has also yielded conflicting results. Therefore, transparency of reporting culture conditions and experimental paradigms may help the field reconcile results. *In vivo* analyses of synaptic dysfunction in C9-ALS/FTD has yet to be fully realized but will become necessary for confirming or denying hypotheses which have been built upon clinical manifestations and *in vitro* models. With all this in mind, the prevailing school of thought is that excitability and synaptic function are aberrant in a temporal fashion in C9-ALS/FTD. The underlying mechanisms of synaptic dysfunction and neuronal excitability continue to be a significant focus of research.

### Patient-derived neuronal models

**Figure 2:**
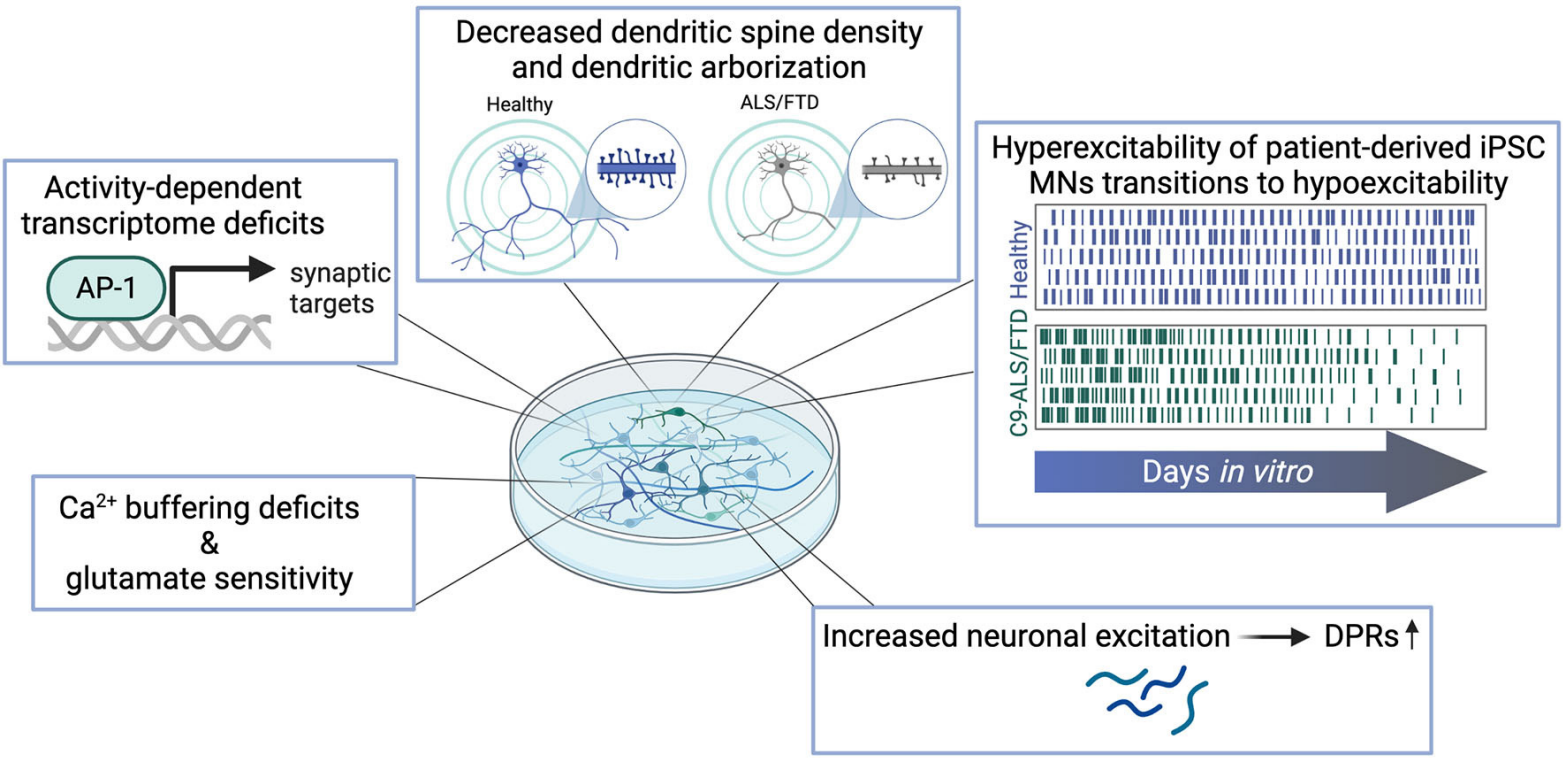
Synaptic dysfunctions observed in patient-derived *in vitro* models of C9-ALS/FTD include Ca^2+^-buffering deficits and increased vulnerability to glutamatergic insult, deficits in the activity-dependent transcriptome, decreased complexity of dendritic arborization and loss of dendritic spines, temporal phases of excitability alterations, and increased DPR levels as a result of hyperexcitation.

Multiple studies have reported changes in neuronal excitability in C9-ALS/FTD patient-derived induced pluripotent stem cell (iPSC) neurons throughout the duration of cell culture *in vitro*. Some of these cultures exhibit hyperexcitability in early stages, which transitions to hypoexcitability in later days of culture (Figure 2) (Wainger et al., 2014; Devlin et al., 2015; Burley et al., 2022). Relatively young C9-ALS/FTD iPSC motor neurons (MNs) display hyperexcitability, namely a higher mean firing rate (Hz) and higher spikes per minute, measured by multi-electrode array (MEA) recordings, an effect that could be reversed by application of retigabine, a voltage-gated potassium (K^+^) channel (Kv7) agonist (Wainger et al., 2014). In this study, the authors ensured MEA recordings were taken only from motor neurons by utilizing a motor neuron-specific fluorescent reporter and purifying using fluorescence-activated cell sorting (FACS). In-line with this, the shift from hyper- to normal or hypo- excitability in C9-NRE iPSC MNs has recently been observed to occur transiently and at early days in culture; hyperexcitability of C9-NRE MNs at earlier stages *in vitro*, with firing rates of control and C9-NRE MNs becoming equal once cultured further (Burley et al., 2022). It is important to note that the iPSC MNs used in this study are not pure MNs; the authors quantify the presence of astrocytes to be ~20% (Burley et al., 2022). Additionally, at the time point at which hyperexcitability was observed in the C9-NRE MNs, this group observed a higher number of neurons double-labeled with Beta-tubulin III and SMI-32, pan-neuronal and motor neuron markers, respectively, which may impact connectivity and excitability in these cultures (Burley et al., 2022). One group reported an increase in the frequency-current (F-I) relationship in C9-NRE iPSC MNs early in culture, but then eventual loss of action potential (AP) firing, without alterations in overall viability of these cultures (Devlin et al., 2015). The cultures used in the Devlin et al. study had similar ratios of motor neurons and astrocytes as in the Burley et al. study, allowing for more direct comparison of these studies. A progressive loss of voltage-gated sodium (Na^+^) and K^+^ currents is also associated with the loss of excitability of these cultures (Devlin et al., 2015). Similarly, under current-clamping conditions, another group showed that C9-NRE iPSC MNs produce fewer spikes with progressive current injection (Sareen et al., 2013). However, these cultures were not assessed over time to pinpoint the timeline of changes in excitability (Sareen et al., 2013). These cultures exhibited similar purity of motor neurons and glial populations (20–30%) as other studies previously described. Sareen et al. (2013) correlated hypoexcitability to RNA sequencing (RNA-seq) results which showed an increase in expression of KCNQ3 (Potassium Voltage-Gated Channel Subfamily Q Member 3) in C9-NRE iPSC MNs. Another study showed that at earlier time points in culture, C9-NRE iPSC MNs do produce more APs with increasing injected current when compared to healthy control MNs (Burley et al., 2022). Conversely, in other studies, no changes in firing properties, F-I relationship, or Na^+^ or K^+^ channel currents were detected in C9-ALS iPSC MNs, which the authors attribute to a lack of glia in their cultures (Selvaraj et al., 2018). Indeed, this group performed a follow-up study investigating non-cell autonomous effects of glia on MN excitability. When healthy MNs were cultured with C9-NRE astrocytes, a progressive loss of action potential firing resulted (Zhao et al., 2020). Once further cultured, healthy motor neurons grown with C9-NRE astrocytes progressively lost Na^+^ or K^+^ channel currents (Zhao et al., 2020). Many groups have shown the potential causal role of ion channel expression or function in mediating temporal excitability in iPSC MNs, and this topic has been recently reviewed (Lorusso et al., 2019). Hyperexcitability has also been observed in C9-NRE iPSC cortical neurons (CNs); an increase in the rate of burst firing (reduction of inter-burst length), measured with the whole-cell patch-clamp technique and MEA recordings (Perkins et al., 2021). Also, an increase in miniature excitatory postsynaptic currents (mEPSCs) without changes in excitatory glutamate receptor response in addition to a reduced ready-releasable pool (RRP) of synaptic vesicles (Perkins et al., 2021). The CNs used in the Perkins et al. study were estimated to be 90–98.5% pure cortical neurons and thus the synaptic dysfunction measured can be attributed to neuronal intrinsic mechanisms (Perkins et al., 2021). Based on present reports, C9-NRE MNs show changes in excitability and ionic currents, in addition to temporal changes in synaptic activity. Currently, only alterations in firing rate, but not changes in ionic currents (Na^+^ and K^+^) have been reported in C9-NRE CNs. To conclude, alterations in excitability of C9-NRE neurons may be intrinsic or non-cell autonomous, as there are studies supporting both postulates.

Deficits in calcium (Ca^2+^) buffering and signaling are known to be associated with neurodegeneration (Marambaud et al., 2009), in addition to glutamate vulnerability (Foran and Trotti, 2009). Although not specific to C9-NRE ALS/FTD, early work has linked cortical hyperexcitability in ALS with disruptions in the excitatory system (Figure 1). Indeed, excess glutamate has been measured in CSF of ALS patients (Rothstein et al., 1990) and low glutamate uptake in the motor cortex, somatosensory cortex, and spinal cord (Rothstein et al., 1992; Bristol and Rothstein, 1996) have been attributed to the loss of the excitatory amino acid transporter 2 (EAAT2) in motor cortex of ALS patients (Bristol and Rothstein, 1996). Alterations in glutamate receptor subunit composition and changes in the properties of these subunits have also been identified in ALS. The RNA-editing enzyme ADAR2, a double-strand RNA-specific deaminase, typically edits the Q/R site of AMPA receptor (AMPAR) subunit GluA2, which dictates the calcium permeability of the AMPAR complex (Kwak and Kawahara, 2005). However, in ALS patient tissue, a loss of RNA-editing at this site makes the AMPARs permeable to calcium and the neurons susceptible to excitotoxicity (Kwak and Kawahara, 2005). Based on evidence from C9-NRE iPSC models, it is not clear that excess synaptic glutamate is responsible for the hyperexcitability observed *in vitro*, and alterations to glutamate receptor composition, function, and expression are not ubiquitously reported. Deficits in this regard are observed in C9-ALS/FTD patient-derived iPSC neurons (Figure 2). Dafinca et al. reported alterations in calcium dynamics via Ca^2+^ imaging in C9-NRE MNs. In this study, C9-NRE MNs have higher Ca^2+^ levels at rest, and potassium chloride (KCl) evokes an increased amplitude of response compared with control iPSC MNs. In contrast, stimulation with glutamate evoked a lesser amplitude of response but induced a prolonged period of Ca^2+^ waves in the diseased motor neurons (Dafinca et al., 2020). The recovery time post-stimulation with KCl and glutamate is delayed in the C9-NRE MNs (Dafinca et al., 2020). These phenotypes were observed in tandem with increased Ca^2+^-permeable GluA3, GluA2, and GluN2A subunits in the C9-NRE MNs (Dafinca et al., 2020). Higher sensitivity to glutamate and thus glutamate-induced excitotoxicity is corroborated by previous studies in C9-NRE MNs (Donnelly et al., 2013; Selvaraj et al., 2018; Shi et al., 2018). Studies employing RNA-seq of C9-NRE MNs found a significant upregulation of GluA1 transcripts and protein levels compared to healthy controls, which the authors found led to a lower threshold for glutamate-induced excitotoxicity (Selvaraj et al., 2018). Additionally, upregulation of GluA1 mRNA in ALS-patient spinal cord tissue, but not frontal cortex, was also observed, corroborating *in vitro* results (Selvaraj et al., 2018). Another study did find increases in GluN1 and GluA1 in postmortem motor cortex (Shi et al., 2018). In C9-NRE MNs at a time point when hyperexcitability is observed, ionomycin provokes a greater delta peak amplitude via Ca^2+^ imaging, which is not observed 7 days later when these cultures are hypoexcitable (Burley et al., 2022), suggesting intracellular calcium release has a role in propagating hyperexcitability. Another study showed that C9-NRE MNs have increased spikes (Ca^2+^ events) per second at baseline (Bursch et al., 2019). Both of these studies also showed deficits in mitochondrial Ca^2+^ buffering; an increase in Ca^2+^ release from mitochondria in C9-NRE MNs upon application of FCCP (carbonyl cyanide-p-trifluoromethoxyphenylhydrazone, a mitochondrial oxidative phosphorylation uncoupler) (Bursch et al., 2019), and a reduction of Ca^2+^ uptake into mitochondria after glutamate stimulation (Dafinca et al., 2020). As calcium is a second messenger, disruptions in intracellular Ca^2+^ levels have clear implications on Ca^2+^-dependent pathways and thus impact many cellular and molecular processes (Marambaud et al., 2009).

### Other models

In addition to patient-derived iPSC neuron models in which potentially all of the pathogenic mechanisms (expression of NRE-RNA, DPRs, and loss of C9orf72) could contribute to phenotypes, there are other models in which only one or two of these pathological phenomena are present. By transducing the NRE-RNA, using artificial constructs to express DPRs, or utilizing C9orf72 knock-out models *in vitro* and *in vivo*, the contribution of each of these pathological mechanisms can be determined separately and in conjunction.

Overall, there are few reports of synaptic dysfunction in rodent models of the C9-NRE. Intriguingly, in a rodent model of C9-ALS/FTD, mice with a bacterial artificial chromosome (BAC), in which there is C9-NRE RNA in addition to DPRs, show loss of interneurons in the spinal cord (Liu et al., 2016) at end-stage points. Corroborating the occurance of synaptic dysfunction in this C9-BAC mouse model, another group reported that before disease onset, there is a decrease in inhibitory signaling in layer V of the motor cortex and increased output of pyramidal neurons in layer V (Amalyan et al., 2022). Conversely, in other C9-NRE BAC mice, there were no differences in excitability, Ca^2+^ transients, or sensitivity to glutamate (O'rourke et al., 2015). In hippocampal neuron cultures, with an expression of 66 x NRE repeats and levels of detectable poly(GP), there was an increased amplitude of response to glutamate and KCl via Ca^2+^ imaging, in addition to increased sensitivity to glutamate-mediated cell death (Huber et al., 2022). Through pharmacological inhibition, the authors determined the glutamate vulnerability was due to extra-synaptic NMDARs (Huber et al., 2022). These deficits correlated with a reduction in dendritic arborization complexity, total dendrite length, the number of branches, and fewer mature dendritic spines (Huber et al., 2022). Dendritic spines are thought to compartmentalize Ca^2+^ (Yuste et al., 2000), and thus, the loss of these compartments may result in an inability to buffer intracellular Ca^2+^. Results in hippocampal cultures could point to aberrations that may tilt the scale more toward FTD clinical phenotypes vs. pure ALS phenotypes.

Electrophysiological properties of neurons are also altered with the expression of DPRs; however, these alterations are not consistently in agreement. In some cases, the expression of DPRs leads to reductions in synaptic transmission and synaptic unloading. In poly(GA)_50_-expressing rat primary cortical neuron cultures, a reduction of synaptic unloading and simultaneous increase in Ca^2+^ influx was reported by Jensen et al., which led to the identification of decreased levels of the synaptic vesicle protein SV2a (Jensen et al., 2020). *In vivo* models of DPR expression also show evidence of electrophysiological alterations. Layer V pyramidal neurons of the prefrontal cortex of 4.5-month-old mice which express poly(GR)_80_ have significantly fewer frequency of mEPSCs without modifications in AMPAR activity (Choi et al., 2019). Conversely, some studies show increased excitability in the presence of DPRs. Poly(PR) exhibits hyperexcitable effects in layer V pyramidal neurons. Motor cortex slices incubated with 100 nM of synthetic poly(PR)_20_ exhibit increased frequency of firing, a reduction in threshold to fire AP (rheobase), and an upward shift in the F-I relationship, which was unique to the motor cortex and not observed in somatosensory or visual cortices (Jo et al., 2022). In this study, Jo et al. determined the cause of hyperexcitability in these experiments to be due to persistent Na^+^ currents, which could be reversed by applying a therapeutic dose of Riluzole or antibodies targeting specific Na^+^ channel subunits (Jo et al., 2022). Expression of poly(PR)_36_ in *Drosophila melanogaster* larvae increases extracellular glutamate and intracellular Ca^2+^ (Xu and Xu, 2018). The discordance of these overall results may be due to DPR species, length, or timing of expression or exposure to the DPRs.

Recently, our laboratory has provided evidence that aberrant neuronal activity and synaptic dysfunction in C9-ALS/FTD is a component of a feedforward loop. In C9-NRE iPSC MN and rat primary cortical and motor neurons, high-frequency stimulation via optogenetics or glutamate bath increases levels of all DPR species (Figure 2) (Westergard et al., 2019). This effect can be diminished by glutamate receptor antagonists (Westergard et al., 2019). Another group recently corroborated our findings, showing that excitatory optogenetic stimulation increases poly(GA) levels in rat primary cortical neurons (Catanese et al., 2021). Furthermore, Catanese et al. showed that the application of Apamin, a Kv7 inhibitor and a compound that inhibits Ca^2+^-activated potassium (SK) channels, can reduce this burden of poly(GA) (Catanese et al., 2021). These potassium channel inhibitors also reduced levels of apoptotic markers in C9-NRE MNs (Catanese et al., 2021), similar to other findings (Wainger et al., 2014). The neurons in this study are likely hyperexcitable due to the use of potassium channel inhibitors, and counterintuitively, this state of neuronal activity was shown to be beneficial. Intriguingly, 1 Hz neuronal inhibition via activation of archaerhodopsin, an outwardly directed proton pump that silences neurons, leads to apoptotic cell death of poly(GA) expressing neurons (Catanese et al., 2021), which suggests a goldilocks dilemma for types of neuronal activity in disease. There is also the possibility that modes of neuronal activity are beneficial or detrimental in a cell-type-specific manner. These latter studies were performed in rat primary cortical neurons, and hyperexcitability *in vitro* has been primarily reported in patient-derived iPSC MNs.

Alterations in synaptic activity and electrophysiological properties due to a loss of C9orf72 protein have also been reported. In zebrafish, reduction of C9orf72 protein leads to reduction of quantal release and frequency of mEPCs (miniature endplate currents) at the neuromuscular junction and delays in synaptic vesicle recycling (Butti et al., 2021). Complete ablation of *C9orf72* leads to deficits in long-term potentiation (LTP) at the dentate gyrus and long-term depression (LTD) at the Schaffer collateral (Ho et al., 2020). LTP and LTD are the most-studied forms of synaptic plasticity that are important in learning and memory (Malenka and Bear, 2004), which are aspects of cognition affected in ALS/FTD. Loss of C9orf72 protein also leads to a reduction in mEPSC frequency in mouse hippocampal cultures (Bauer et al., 2022). In the cortex of C9 knockout mice, there are increased levels of the AMPAR subunit GluA1, which the authors link to enhanced excitotoxicity (Xiao et al., 2019). C9-NRE iPSC MNs and C9 knockout iPSC MNs, which both have reduced levels of C9orf72 protein, are more sensitive to glutamate excitotoxicity and have increased surface expression of GluN1 (Shi et al., 2018). This phenotype can be rescued by the replacement of C9orf72 protein or the use of retigabine, an activator of the voltage-gated potassium channel, Kv7, (Shi et al., 2018), which has been previously shown to promote C9-NRE iPSC MNs survival and reduce hyperexcitable cultures (Wainger et al., 2014).

Isolating each pathological mechanism of C9-ALS/FTD still results in synaptic dysfunction; however, it is important to note that the expression of NRE-RNA may allow RAN translation to occur. Therefore, DPRs may also be present in this type of model.

## Regulation and alteration of synaptic proteins

Due to the alterations in overall synaptic function associated with the C9 mutation, it is conceivable that C9orf72 has a direct or indirect synaptic function. Indeed, the C9orf72 protein is found to have a synaptic localization (Frick et al., 2018; Xiao et al., 2019), but its precise role at the synapse is not yet clear. C9-ALS/FTD *in vitro* models show aberrations in synaptic transcripts and protein levels in addition to morphological defects. This section highlights the implication of the C9-NRE, DPRs, and loss of C9orf72 protein on gross morphological phenotypes and synaptic proteins.

### Patient-derived neuronal models

Models of C9-ALS/FTD have shown alterations in several cellular pathways, such as nucleocytoplasmic transport, proteostatic imbalance, RNA metabolism, and inflammation (Haeusler et al., 2016; Balendra and Isaacs, 2018; Cicardi et al., 2021), but synaptic pathways and gene networks have been consistently identified as aberrant. From RNA-seq experiments on C9-NRE MNs, there was upregulation of synaptic proteins Dipeptidyl Peptidase Like 6 (DPP6) and its interactor, Potassium Voltage-Gated Channel Subfamily Q Member 3 (KCNQ3), as well as cerebellin family proteins CBLN1, CBLN2, and CBLN4 (Sareen et al., 2013). One such synaptic transcript, CBLN1 was also recently identified to be downregulated in C9-NRE CNs, indicating potential divergence in synaptic components (Perkins et al., 2021). Alternatively, another study that performed RNA-seq on C9-NRE MNs revealed via gene ontology analysis that mRNAs encoding synaptic proteins are downregulated in C9-NRE MNs (Selvaraj et al., 2018). These synaptic mRNAs include KCNJ2, an inwardly rectifying potassium channel, synaptotagmin-5 (SYT5), and NMDAR subunit GRIN3B (Selvaraj et al., 2018). In a recent transcriptomics study of C9-NRE iPSC MNs, an analysis of potential transcriptional regulators of the differentially expressed transcripts showed that the transcripts that are upregulated in C9-NRE MNs are likely driven by the AP-1 transcription factor complex, which is comprised of FOS and JUN heterodimers (Neuro et al., 2021) and is known to be regulated by neuronal activity or other stimuli (Kaminska et al., 2000). Given that increased usage of the AP-1 transcription factor complex could be reflective of a hyperexcitable state, regulation and maintenance of synapses could be directly affected by overactivation of the activity-dependent transcription pathway. Based on the detailed case for hyperexcitability presented for C9-ALS/FTD earlier in this review, investigations into alterations in the activity-dependent transcriptome within patient-derived cells are of interest and an active avenue of research in our laboratory and others. A recent study showed age-dependent dysregulation of the activity-dependent transcription factor, cAMP/Ca^2+^ response element-binding protein (CREB) in C9-NRE iPSC MNs (Catanese et al., 2021). In the early stages, there were elevated levels of phosphorylated (active) CREB in the C9-NRE iPSC MNs, which matched the hyperexcitable state of the culture (Catanese et al., 2021). This transitioned to a loss of CREB activity when the cultures became hypoexcitable (Catanese et al., 2021). These phenomena were also observed in rat primary cortical neurons expressing poly(GA)_175;_ at early stages of culture there was increased p-CREB, with progressive loss of p-CREB when cultured further. The loss of CREB activity correlated with the downregulation of synaptic genes, including synaptotagmin-1 and neuroligin-3, and also a loss of synapses (Catanese et al., 2021). Inhibition of Kv7 and SK potassium channels were found to be neuroprotective, and restored CREB transcriptional activity and levels of synaptic RNAs regulated by CREB (Catanese et al., 2021). This study provides further evidence of dysregulation of the activity-dependent transcriptome in ALS/FTD neurons. Patient-derived cortical neurons also show defects in synaptic transcripts, as RNA-seq revealed a reduction of Protocadherin Gamma Subfamily C 4 (PCDHGC4), which is a synaptic protein that negatively regulates neuroligin-1, and thus controls synapse number, and indeed these neurons had an increased excitatory synaptic density; increase in colocalized pre-synaptic marker synapsin-1 and post-synaptic marker PSD-95 (Perkins et al., 2021).

### Other models

In addition to alterations in synaptic transcripts and protein levels, the gain-of-function (NRE-RNA, DPRs) and loss-of-function (loss of C9orf72 protein) mechanisms of the C9-NRE lead to defects in synaptic morphology, namely a loss of synapses and loss of dendritic arborization complexity. In rat primary cortical and motor neuron cultures, in conjunction with poly(GA) overexpression, a loss of the synaptic vesicle protein, SV2a, was observed (Jensen et al., 2020). This loss of SV2a was also seen when animals expressing poly(GA)_149_ were examined in the spinal cord and at the NMJ (Jensen et al., 2020). Mice that express poly(GR)_80_ under a CamKII promoter postnatally undergo DPR accumulation in an age-dependent manner (Choi et al., 2019). In these mice at 6 months of age, an age where poly(GR) starts to accumulate, there is a significant reduction in PSD-95 protein in cortical regions (Choi et al., 2019). *Drosophila melanogaster* expressing either C9-NRE repeats or DPRs show decreased dendritic arborization complexity and reduced synaptic boutons (Burguete et al., 2015; Perry et al., 2017; Park et al., 2020). This phenomenon has been corroborated in mice (May et al., 2014). More severe deficits in dendritic branching are seen with arginine-containing DPRs and neuritic localization of the NRE-RNA, suggesting a more drastic effect at synaptic sites (Burguete et al., 2015; Park et al., 2020). At the NMJ, the expression of NRE-RNA reduces the number of NMJ active zones and synaptic boutons in fly larvae (Freibaum et al., 2015; Zhang et al., 2015).

Furthermore, as C9orf72 is known to be localized to synaptic regions (Xiao et al., 2019), there is the possibility that C9orf72 has a function at the synapse. Strengthening this argument, partial or complete ablation of C9orf72 has consequences on synaptic proteins and structure, in addition to electrophysiological properties discussed above. Loss of C9 protein leads to a reduction in localization of pre-and post-synaptic markers at the NMJ of zebrafish and a reduction of synapse size (Butti et al., 2021). In C9-knockout (C9-KO) mouse cortex, there is a loss of complexity of dendrite arborization, a reduction in the total length of the dendrites, and a reduction of spine density (Ho et al., 2019; Lall et al., 2021). A drop in synaptic Rab proteins, such as Rab39b, is also observed in the C9-KO mouse cortex (Xiao et al., 2019). Most recently, knockdown of C9orf72 protein led to a reduction in excitatory synapses in primary rat hippocampal cultures (Bauer et al., 2022). In this study, loss of C9orf72 protein levels was concordant with loss of synapsin-1, synapsin-3 and SV2 (Bauer et al., 2022). In a proteomics screen, the C9orf72 protein interacted with cofilin, a protein that regulates the actin cytoskeleton (Sivadasan et al., 2016). Dendritic spines are composed primarily of actin (Hering and Sheng, 2001), therefore, C9orf72 may play a role in regulating dendritic spine dynamics through these interactions. Disruptions in the C9orf72-cofilin interaction may lead to deficits in dendritic branching and dendritic spine composition. Indeed, alterations in dendritic spines in relation to the C9-NRE have been observed as described (May et al., 2014; Burguete et al., 2015), and in neurites, the C9-NRE repeats can be bound by the activity-regulated RNA-binding protein, Fragile-X mental retardation protein (FMRP) and are neuritically localized (Burguete et al., 2015). Thus, the intronic region of the C9orf72 gene may serve as a synaptic localization signal. Along these lines, C9orf72 has been shown to interact with synaptic proteins SV2a (Butti et al., 2021), PSD-95 (Xiao et al., 2019), and the synapsin family of proteins (Bauer et al., 2022), in addition to endosomes (Shi et al., 2018), and regulates lysosomal biogenesis in motor neurons further highlighting the potential role of C9orf72 protein at the synapse. NRE-RNAs or DPRs at synaptic sites may also impair local neuritic or synaptic translation, thus inhibiting cytoskeletal remodeling and maintenance. A reduction in axon length was also observed due to the knockdown of C9 protein (Sivadasan et al., 2016). The authors of this study link the role of C9orf72 in the endosome-lysosome pathway to increased expression of glutamate receptors. In the motor cortex of C9-KO mice, loss of C9 protein in microglia leads to enhanced synaptic pruning, an increase in complement cascade factor C1q, detectable synaptophysin signal engulfed by microglia, and a loss of vGlut1 and synaptophysin (Lall et al., 2021). It is important to note that while C9orf72 protein is localized at the synapse, it also plays are role in membrane trafficking and autophagic regulation, and this role has recently been reviewed elsewhere (Tang, 2016). Thus, whether or not the synaptic deficits observed with the loss of C9orf72 is direct or indirect has yet to be seen. Considering all of these studies, the C9-NRE, DPRs, and loss of C9orf72 lead to aberrant excitability detailed in the section above, broadly changing the synaptic landscape.

## Discussion and conclusion

Communication breakdown at the synaptic level has been observed in many genetic and sporadic forms of ALS and FTD. These breakdowns include electrophysiological alterations, excitatory and inhibitory neurotransmitter system defects, such as glutamate or GABA receptor subunit expression, pre- and post-synaptic protein expression, and ion channel dysregulation. In addition to the reported C9orf72-linked cortical hyperexcitability observed early in the disease timeline (Geevasinga et al., 2015; Wainger and Cudkowicz, 2015; Schanz et al., 2016), a few case studies have revealed that epilepsy can co-occur with C9-FTD (Melis et al., 2022), and C9-ALS (van den Ameele et al., 2018). Epilepsy is a manifestation of synchronous or excessive neuronal activity (Devinsky et al., 2018) and thus intersects with what is known regarding excitability in ALS/FTD. While this review has focused on the synaptic dysfunctions associated with the C9-NRE, synaptic deficits are also observed in other genetic forms of ALS/FTD. Synaptic dysfunction as a result of SOD1 mutations is widely reported (Kuo et al., 2005; Bories et al., 2007; Pieri et al., 2009; Magrane et al., 2012). Mutations in TDP-43, in addition to nuclear loss of TDP-43, can lead to a myriad of synaptic dysfunctions which vary based on region of mutation (Fogarty et al., 2016; Chand et al., 2018; Dafinca et al., 2020; Dyer et al., 2021; Ni et al., 2021). Similarly, mutations in FUS and/or nuclear loss of FUS leads to changes in excitability and synaptic function (Armstrong and Drapeau, 2013; Sephton et al., 2014; Sahadevan et al., 2021; Scekic-Zahirovic et al., 2021). Overall, it has become clear that genes causative of ALS and FTD, including C9orf72, either have a function at the synapse or are associated with altered synaptic function when mutated. Changes in synaptic morphology, function, and expression of synaptic proteins are often an early observed phenomenon; it would be advantageous to determine mechanistic events that perpetuate synaptic dysfunction to develop treatments that can be used in a therapeutic window.

An upstream event that may perpetuate disease progression is alterations in the activity-dependent transcriptome (Figure 2). It could be that mutations in ALS/FTD genes of interest cause changes in transcription factor complex binding, levels of transcription factor proteins, or activity-dependent epigenetic modifications, therefore activating or suppressing genes required for synaptic maintenance or remodeling. There is also the potential that the pathway is hyper- or hypo-activated due to aberrant activity or protein aggregates observed in patients. Indeed, it is known that in Alzheimer's disease (AD), the activity-dependent transcription factor cAMP/Ca^2+^ response element-binding protein (CREB) is downregulated in patient brain tissue and is inversely related to amyloid-beta plaques, one of the significant hallmarks of the disease (Pugazhenthi et al., 2011). Furthermore, targets of CREB, which are linked to cognition and memory, are reduced and are proposed to be mediated by the amyloid-beta aggregates (Espana et al., 2010). Indeed, a recent study identified age-dependent CREB dysfunction in C9-NRE iPSC MNs (Catanese et al., 2021). Other activity-dependent pathways, such as regulation of local synaptic translation (Holt et al., 2019), could also be impacted by protein aggregation and aberrant neuronal activity.

Synaptic dysfunction in ALS and FTD presents a causality dilemma. It is currently unknown if synaptic dysfunction is causal to neuron death in ALS and FTD or if neurodegeneration causes synaptic dysfunction. There is also the possibility that a triggering event can induce a feed-forward loop of neurodegeneration and synaptic dysfunction in sequence. Clinical evidence shows us that aberrations in neuronal activity (cortical hyperexcitability) may be an early event or at least occurs before significant degeneration of the corticospinal tract (Vucic et al., 2008). Still, it is unclear what triggers this initial event, if it is an early event. Identifying the chicken and the egg in the scenario of synaptic dysfunction in ALS and FTD will become essential.

In summary, morphological defects, functional defects, neurotransmitter receptor changes, as well as changes in synaptic mRNAs and proteins are associated with the neurodegenerative diseases ALS and FTD. Neurons are the ultimate cell type affected in these diseases. Thus, the features which define neurons: synaptic connections, and chemical and electrical activities, are valuable modalities worth focusing current and future research on.

## Author contributions

LG was the lead individual on review conceptualization and execution and was the primary author of the manuscript. AH and DT were consulted for review scope and writing contributions. BJ led review conceptualization, provided supervision, and was the secondary contributor to writing and editing the manuscript. All authors read and approved the final manuscript.

## Funding

This work was supported by the following sources: RF1NS114128 to LG and AH, RF1-AG057882 and R21-NS0103118 and the Muscular Dystrophy Association to DT and BJ.

## Conflict of interest

The authors declare that the research was conducted in the absence of any commercial or financial relationships that could be construed as a potential conflict of interest.

## Publisher's note

All claims expressed in this article are solely those of the authors and do not necessarily represent those of their affiliated organizations, or those of the publisher, the editors and the reviewers. Any product that may be evaluated in this article, or claim that may be made by its manufacturer, is not guaranteed or endorsed by the publisher.
